# Survey-Reported Coverage in 2019-2022 and Implications for Unwinding Medicaid Continuous Eligibility

**DOI:** 10.1001/jamahealthforum.2024.0430

**Published:** 2024-04-05

**Authors:** Adrianna McIntyre, Rebecca B. Smith, Benjamin D. Sommers

**Affiliations:** 1 Department of Health Policy and Management, Harvard T.H. Chan School of Public Health, Boston, Massachusetts; 2 Department of Medicine, Brigham and Women’s Hospital, Harvard Medical School, Boston, Massachusetts

## Abstract

**Importance:**

Policy changes and the COVID-19 pandemic affected health coverage rates, and the “unwinding” of Medicaid’s continuous coverage provision in 2023 and 2024 may cause widespread coverage loss. Recent coverage patterns in national survey and administrative data can inform these issues.

**Objective:**

To assess national and state changes in survey-based Medicaid, private insurance, and uninsured rates between 2019 and 2022, as well as how these changes compare with administrative Medicaid enrollment totals.

**Design, Setting, and Participants:**

This cross-sectional study analyzes nationally representative survey data for all US residents in the American Community Survey (ACS) from 2019 to 2022 compared with administrative data on Medicaid and the Children’s Health Insurance Program from the Centers for Medicare & Medicaid Services (CMS). Data analysis was conducted between June 2023 and January 2024.

**Exposures:**

The COVID-19 pandemic, the Medicaid continuous coverage provision, and policy efforts to increase Marketplace coverage.

**Main Outcomes and Measures:**

Medicaid coverage (self-reported [ACS] and administratively recorded [CMS]), survey-reported uninsured, Medicare, and private insurance status.

**Results:**

A nationally representative sample consisted of 12 506 584 US residents of all ages (survey-weighted 59.7% aged 19-64 years and 50.6% female). CMS statistics showed an increase in Medicaid coverage of 5.2 percentage points as a share of the population from 2019 to 2022. However, changes in the uninsured rate and survey-reported Medicaid were smaller: −1.2 (95% CI, −1.3 to −1.2) percentage points and 1.3 (95% CI, 1.2-1.4) percentage points, respectively. There was a 3.9 percentage point increase in the ACS’s “undercount” of Medicaid enrollment, compared with CMS data, from 2019 to 2022. This undercount was larger among children than adults but smaller in states that recently expanded Medicaid. Rates of additional forms of coverage (such as private insurance) among those in Medicaid also grew during this time.

**Conclusion and Relevance:**

In this cross-sectional study, the uninsured rate declined considerably from 2019 to 2022 but was just one-fourth as large as the growth in administrative Medicaid enrollment under the pandemic continuous coverage provision. Survey-based Medicaid growth was far smaller than administrative growth. This suggests that many people who remained enrolled in Medicaid during the pandemic did not realize that their coverage had continued. These findings have implications for projecting uninsured changes during unwinding, as well as the effect of continuous coverage policies on continuity of care.

## Key Points

**Question:**

How did survey-based coverage rates change during the COVID-19 pandemic, and how did these changes compare with growth in administrative Medicaid enrollment during this period?

**Findings:**

In this cross-sectional study, administrative statistics showed an increase in Medicaid coverage between 2019 and 2022 of 5.2 percentage points as a share of the population. However, changes in the uninsured rate and survey-reported Medicaid were much smaller (−1.2 and 1.3 percentage points, respectively) during this same period.

**Meaning:**

Many people who remained enrolled in Medicaid under the federal continuous coverage provision during the pandemic did not realize that their coverage had continued.

## Teaser

This cross-sectional study assesses national and state changes in survey-based Medicaid, private insurance, and uninsured rates between 2019 and 2022, as well as how these changes compare with administrative Medicaid enrollment totals.

## Introduction

Medicaid enrollment grew to a historic high during the COVID-19 pandemic, as states kept enrollees continuously covered by Medicaid in exchange for enhanced federal funding.^1,2^ Congress also passed the American Rescue Plan, which expanded the generosity and availability of tax credits for Marketplace coverage in 2021 (later extended by the Inflation Reduction Act),^3^ and the Biden administration increased outreach efforts for Medicaid and the Marketplace.^4^ Finally, the economy rebounded from the pandemic-associated recession, which had led to large temporary increases in unemployment. By 2022, survey data indicated that the US had reached its lowest-ever uninsured rate.^5^

However, the Medicaid continuous coverage requirement ended April 1, 2023; by mid-2024, states are expected to finish redetermining eligibility for all enrollees whose coverage was maintained under the requirement. As of February 2024, more than 16 million people have been removed from the program in the “unwinding” of this provision, with wide variation by state.^6,7^

At the same time, research shows that Medicaid enrollment growth (as measured by enrollment records) in 2021 exceeded survey-reported rates of Medicaid coverage, raising questions about how beneficiaries understood their coverage during the pandemic and during the unwinding.^8^ Survey data are critical to understanding how beneficiaries perceive their coverage and also offer information on alternative coverage (such as employer-sponsored insurance [ESI]) and uninsured status that are not captured in Medicaid administrative data.

The objective of this cross-sectional study was to use recently released national data from the American Community Survey (ACS) to examine national and state changes in coverage through the end of 2022 and to compare self-reported changes in Medicaid and uninsured rates from national surveys to administrative Medicaid enrollment totals. These results have important implications for understanding Medicaid enrollees’ perceived coverage status entering the 2023 expiration of the continuous coverage provision and how the unwinding process itself may ultimately affect survey-based uninsured estimates in 2024 and beyond.

## Methods

### Overview

This observational study used the US Census Bureau’s ACS to estimate national and state-level changes in self-reported coverage through Medicaid, Medicare, private insurance, and uninsured rates from 2019 to 2022 (the most recent year of available ACS data). Data analysis was conducted between June 2023 and January 2024. This study meets the Strengthening the Reporting of Observational Studies in Epidemiology (STROBE) reporting guidelines for cross-sectional studies^9^ and was deemed not human participant research by Harvard University’s institutional review board.

### Data Sources

The ACS is the nation’s largest household survey and the primary source for state-specific estimates of self-reported health coverage (see the eMethods in Supplement 1 for exact question wording). We used the ACS Public Use Microdata Sample.^10^ The study also used contemporaneous administrative data from the Centers for Medicare & Medicaid Services (CMS)^2^ to measure changes in the average monthly share of each state’s population covered by Medicaid or the Children’s Health Insurance Program. CMS data include state monthly totals across all age groups and national totals (but not state-level data) for children vs adults from the monthly enrollment reports.^11,12^ State population totals were from the ACS. The 2020 ACS was affected by pandemic shutdowns, and the US Census Bureau advises against using these data for direct comparisons with other survey years^13^; accordingly, we included 2020 results in the initial descriptive statistics, but all other comparisons of coverage over time excluded 2020 and were instead based on 2 periods: 2019 to 2021 and 2021 to 2022, which are years not affected by data quality concerns.

### Statistical Analysis

First, we examined national changes in Medicaid, Medicare, private insurance, and uninsured rates based on survey data. We examined both the full population (which is most directly comparable to enrollment statistics from CMS, which include adults 65 years and older) and by age group (children, the largest single enrollment group in Medicaid; working-age adults, for whom employment-related coverage changes are more common; and adults 65 years and older, who are overwhelmingly enrolled in Medicare). Then, we compared nationwide 2019 to 2022 changes in self-reported coverage to CMS administrative Medicaid enrollment, for the full population and then separately for children (ages 0-18 years) and adults (19 years and older), to match the age breakdown publicly available in monthly CMS data.

Second, we estimated state-level linear regressions to explore associations between changes in survey-reported coverage and administrative Medicaid enrollment for all 50 states and Washington, DC. The outcomes for these 2 regression models were the state-level change in the survey-reported shares of the population with (1) Medicaid and (2) without any health insurance between 2019 and 2022. The independent variable in both models was the state change in administrative Medicaid enrollment over the same time period. These associations may be affected by whether and when a state expanded Medicaid under the Affordable Care Act; in particular, beneficiaries may be more likely to be aware of their coverage status if they recently enrolled in a state’s recent Medicaid expansion, compared with maintaining previous enrollment during the pandemic. Accordingly, we stratified by timing of Medicaid expansion (recent expansions, 2019-2022; older expansions, before 2019; and nonexpansion, as of 2022). Regressions were weighted by state population and used statistical significance thresholds of *P* < .05.

Third, we assessed survey-reported changes in Medicaid and uninsured rates from 2019 to 2022 by age, income (based on the family health insurance unit^14^), and race and ethnicity (self-reported in the ACS). Subgroup estimates are presented to assess potential heterogeneity in coverage changes and worsening or improving disparities. We also examined subgroups for whom loss of Medicaid eligibility would be more common than other populations in the absence of the continuous coverage provision, and who therefore may have been able to remain in Medicaid at higher rates from 2021 to 2022 than previous years (eMethods in Supplement 1).

Finally, since coverage outcomes after the unwinding are likely to be influenced by alternative insurance options available to people leaving Medicaid, we assessed what share of people reporting Medicaid coverage each year also reported having other insurance in the same year. We examined Medicare, nongroup private insurance (including Marketplace coverage), ESI, or any private insurance, as the ACS allows people to indicate 1 or more types of health insurance coverage. Medicaid enrollees are permitted to carry additional sources of insurance coverage; in these cases, the program typically acts as a secondary payer, or the “payer of last resort.”^15^ All analyses were conducted in Stata, version 16 (StataCorp).

## Results

The sample included 12 506 584 individuals, representative of the US population (eTable 1 in Supplement 1). The self-reported uninsured rate declined by 0.6 percentage points between 2019 and 2021 and 0.6 percentage points from 2021 to 2022 (Table 1). The decline in the uninsured rate was largest for adults aged 19 to 64 years (−1.7 percentage points total from 2019-2022), with smaller changes for children (−0.6 percentage points) and no change for adults 65 years and older. While self-reported Medicaid grew substantially between 2019 and 2021 (1.3 percentage points for the full population), it grew just 0.1 percentage point from 2021 to 2022. Coverage gains from 2021 to 2022 for working-age adults were largely driven by a 0.8 percentage point increase in private insurance (compared with just 0.2 for Medicaid in this age group).

**Table 1.** Survey-Reported vs Administrative Changes in Health Coverage Before and During the COVID-19 Public Health Emergency, 2019-2022^a^

Year	%				
	Administrative data, Medicaid	Survey data (n = 12 506 584)			
		Medicaid	Private insurance	Medicare	Uninsured
**Full US population**					
2019	21.8	20.1	66.9	18.2	9.4
2020^b^	20.8	20.1	67.5	18.5	9.1
2021	25.2	21.3	66.6	18.3	8.9
2022	27.0	21.4	66.8	18.7	8.2
Net change, 2019-2021	3.4	1.2	−0.3	0.1	−0.6
Net change, 2021-2022	1.8	0.1	0.2	0.4	−0.6
Net change, 2019-2022	5.2	1.3	−0.1	0.5	−1.2
**Ages 0-18 y**					
2019	45.3	37.6	60.3	0.6	5.7
2020^b^	47.4	37.5	61.7	0.6	5.5
2021	50.3	39.1	60.4	0.6	5.4
2022	53.3	39.1	60.7	0.6	5.1
Net change, 2019-2021	5.1	1.5	0.1	0.0	−0.3
Net change, 2021-2022	3.0	−0.1	0.3	0.0	−0.3
Net change, 2019-2022	8.1	1.5	0.4	0.0	−0.6
**Ages 19-64 y**					
2019	NA^c^	14.6	72.2	3.8	13.3
2020^b^		15.0	72.6	3.7	12.8
2021		16.2	72.1	3.6	12.5
2022		16.4	72.9	3.4	11.6
Net change, 2019-2021		1.6	−0.1	−0.3	−0.8
Net change, 2021-2022		0.2	0.8	−0.1	−0.9
Net change, 2019-2022		1.8	0.7	−0.4	−1.7
**Ages ≥65 y**					
2019	NA^c^	14.9	57.3	95.8	0.8
2020^b^		14.3	57.2	95.9	0.8
2021		14.9	55.5	95.4	0.8
2022		15.2	54.0	95.3	0.9
Net change, 2019-2021		0.0	−1.8	−0.4	0.0
Net change, 2021-2022		0.3	−1.4	−0.1	0.0
Net change, 2019-2022		0.3	−3.3	−0.5	0.0

Abbreviation: NA, not applicable.

^a^ Estimates are share of the US population with various forms of coverage (administrative and self-reported). Self-reported coverage data are from the American Community Survey. Administrative data are average monthly enrollment from the Centers for Medicare & Medicaid Services; the share is generated using annual national population estimates (all ages) from the American Community Survey. Numbers may not sum exactly due to rounding.

^b^ Data from 2020 were affected by nonresponse bias and other quality issues noted by the US Census Bureau during the pandemic’s first year.

^c^ Data distinguishing monthly enrollment by age group were only available for adults vs children, so those 19 to 64 years vs 65 years and older were not able to be compared.

**Figure 1.**
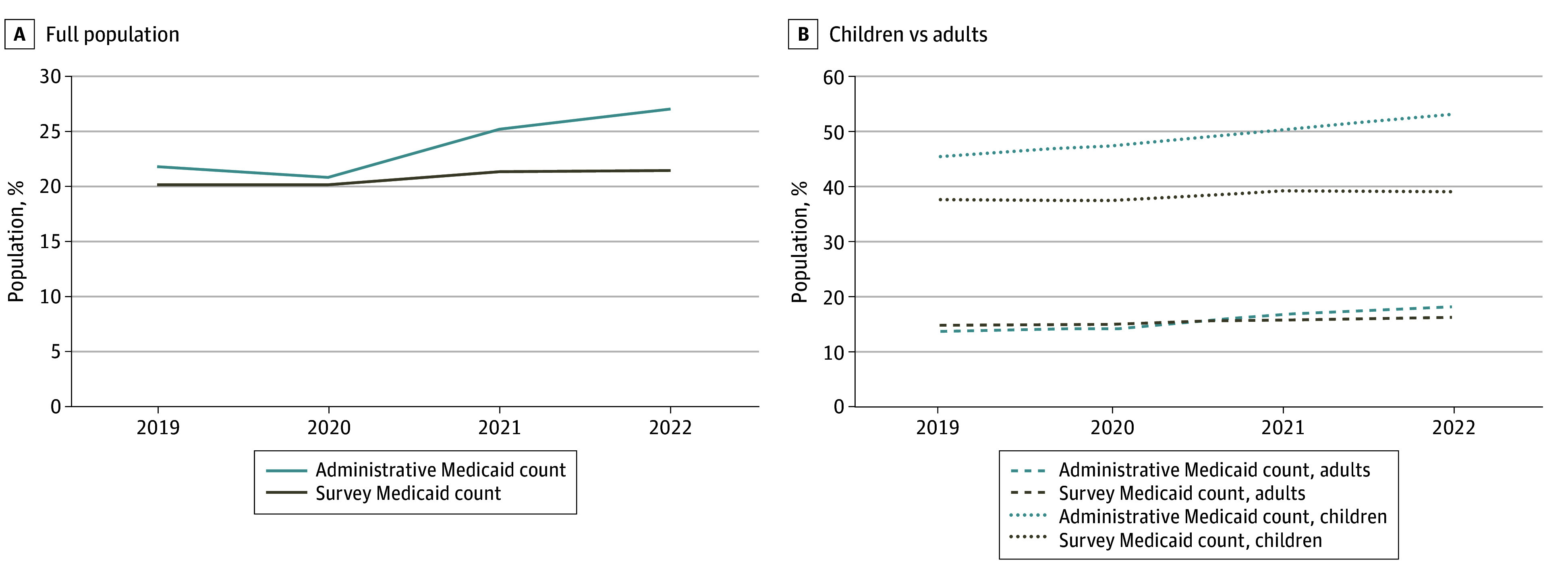
Administrative and Survey Data on Medicaid Coverage Before and During the COVID-19 Public Health Emergency, 2019-2022 This figure shows the share of the US population with Medicaid coverage (administrative and self-reported). Self-reported coverage data are from the American Community Survey. Administrative estimates use Centers for Medicare & Medicaid Services enrollment data; the share is generated using annual national population estimates (all ages) from the American Community Survey. Note that data from 2020 were affected by nonresponse bias and other quality issues noted by the US Census Bureau during the pandemic’s first year.

Table 1 and Figure 1A show that official Medicaid enrollment in CMS data, as a share of the total population, grew 5.2 percentage points from 2019 to 2022, while self-reported Medicaid coverage only grew 1.3 percentage points. This increased the gap—or undercount—between administrative and self-reported Medicaid from 1.7% of the population in 2019 to 5.6% in 2022. The growth in this gap was much larger for children, for whom it increased by 6.5 percentage points, than for adults, for whom it increased by 3.0 percentage points—actually going from a slight overcount to an undercount (Figure 1B). In 2019, only 34 states and Washington, DC had a survey Medicaid undercount, which ranged from 0.8 to 11.2 percentage points; by 2022, all 50 states and Washington, DC had an undercount, ranging from 1.3 to 19.0 percentage points (eTable 2 in Supplement 1).

**Figure 2.**
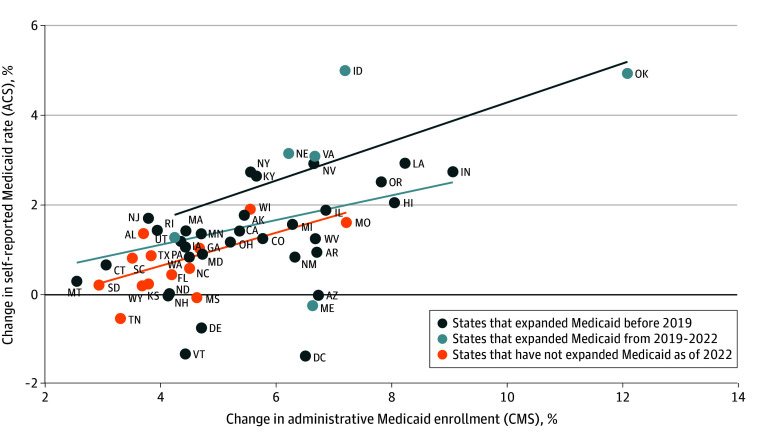
State-Level Association Between Changes in Administrative Medicaid Enrollment Rates and Self-Reported Medicaid Rates (2019 vs 2022) by Medicaid Expansion Timing Changes refer to state-level percentage point changes between 2019 and 2022 in each outcome. Lines are the fitted linear regression estimates for a state-level model (n = 51, for all states plus Washington, DC), and regressions are weighted by state population. Regression coefficients were as follows: recent expansion states, β, 0.44 (95% CI, 0.23-0.65); older expansion states, β, 0.28 (95% CI, 0.12-0.43); and nonexpansion states, β, 0.37 (95% CI, 0.08-0.66). ACS indicates the American Community Survey; CMS, Centers for Medicare & Medicaid Services.

Figure 2 plots state-level changes in administrative Medicaid enrollment against changes in survey-reported Medicaid. These 2 measures were more positively associated in newly expanding states (regression coefficient β, 0.44; 95% CI, 0.23-0.65; *P* = .004) than in other expansion states (β, 0.28; 95% CI, 0.12-0.43; *P* = .001); the association in nonexpansion states fell in between but was less precisely estimated (β, 0.37; 95% CI, 0.08-0.66; *P* = .02). This means that each 1.0 percentage point gain in Medicaid in CMS data was linked to a 0.44 percentage point gain in survey-reported Medicaid in new expansion states, and 0.28 and 0.37 in older expansion and nonexpansion states, respectively, indicating that well below half and in some cases less than one-third of administrative coverage gains registered in survey-reported Medicaid gains.

The eFigure in Supplement 1 plots state-level changes in administrative Medicaid enrollment against changes in the survey-reported uninsured rate. The point estimate showed the strongest association for recent expansion states (β, −0.21; 95% CI, −0.32 to −0.10; *P* = .006). Older expansion states had a slightly weaker association (β, −0.18; 95% CI, −0.32 to −0.03; *P* = .02), while nonexpansion states showed no statistically significant association between Medicaid enrollment gains and uninsured changes (β, 0.03; 95% CI, −0.32 to 0.33; *P* = .99). Overall, these regression coefficients indicate that the ratio of administrative Medicaid coverage gains to uninsured decline was roughly 5:1, meaning that approximately 80% or more of the pandemic-era Medicaid enrollment gains did not produce commensurate reductions in the self-reported uninsured rate.

Table 2 summarizes survey-based Medicaid changes over 2 time frames: from 2019 to 2021 (as the continuous coverage provision took effect in March 2020), and then from 2021 to 2022 (the last full year of the continuous coverage provision). Medicaid changes between 2019 and 2021 were larger than between 2021 and 2022. For the overall population, survey-reported Medicaid was stable between 2021 (21.3%) and 2022 (21.4%), even as administrative enrollment statistics indicated a 1.8 percentage point increase in Medicaid coverage as a share of the population. This general pattern held for most race, ethnicity, and age groups, with changes in self-reported Medicaid from 2021 to 2022 only exceeding 0.5 percentage points for Black individuals (0.7 percentage points) and American Indian and Alaska Native individuals (1.7 percentage points). Notably, self-reported Medicaid did not increase at all between 2021 and 2022 for children, even though administrative enrollment among children grew by 3.0 percentage points during this period.

**Table 2.** Medicaid Coverage Changes for Select Subgroups, 2019-2022 (N = 12 506 584)^a^^,^^b^

Group	%				
	Medicaid		2021 vs 2019, pp change	2022 Medicaid	2022 vs 2021, pp change
	2019	2021			
Full population (administrative data)	21.8	25.2	3.4	27.0	1.8
Full population (survey)	20.1	21.3	1.2	21.4	0.1
Race and ethnicity (survey)					
American Indian and Alaska Native (non-Hispanic)	33.2	36.9	3.7	38.6	1.7
Asian American, Native Hawaiian, and Pacific Islander (non-Hispanic)	16.3	18.4	2.1	18.0	−0.4
Black (non-Hispanic)	32.0	33.3	1.3	34.0	0.7
Hispanic	30.0	31.1	1.1	31.2	0.1
White (non-Hispanic)	14.4	15.5	1.1	15.4	−0.1
Age group (survey), y					
0-18	37.6	39.1	1.5	39.1	0.0
19-64	14.6	16.2	1.6	16.4	0.2
≥65	14.9	14.9	0.0	15.2	0.3
Income as percentage of the FPL (survey)^c^					
0%-138%	48.3	49.3	1.0	50.1	0.8
139%-250%	23.5	26.9	3.4	28.4	1.5
251%-400%	9.5	12.1	2.6	12.9	0.8
>400%	3.4	4.6	1.2	4.6	0.0

Abbreviations: FPL, federal poverty level; pp, percentage point.

^a^ Estimates are the share of the US population with Medicaid coverage (administrative and self-reported). Self-reported coverage data are from the American Community Survey. Administrative data are from the Centers for Medicare & Medicaid Services; the share is generated using annual national population estimates (all ages) from the American Community Survey.

^b^ Data from 2020 were affected by nonresponse bias and other quality issues noted by the US Census Bureau during the pandemic’s first year; therefore, those results are not included in estimates of changes over time.

^c^ Income was defined by the health insurance unit as a percentage of the FPL.

Self-reported Medicaid coverage was most common in the lower-income range, with approximately half of those with incomes under 138% of the federal poverty level (FPL) reporting Medicaid coverage. However, gains in self-reported Medicaid were largest for those in the income range of 139% to 250% of the FPL, with a 3.4 percentage point gain between 2019 and 2021 and a 1.5 percentage point gain between 2021 and 2022. Compared with the population average, survey-reported Medicaid also increased more substantially for several groups that are more likely to have experienced eligibility changes—namely those with incomes near the threshold for their eligibility group or those potentially losing eligibility after aging out of childhood eligibility (eTable 3 in Supplement 1).

**Figure 3.**
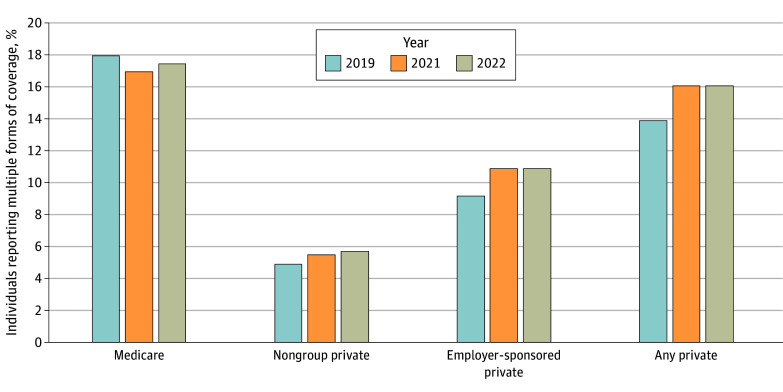
Survey-Reported Rates of Multiple Forms of Coverage Among those Reporting Medicaid Coverage, 2019-2022 This figure shows the percentages of those reporting Medicaid coverage who also reported having another form of coverage. All data are from the American Community Survey. Data from 2020 were affected by nonresponse bias and other quality issues noted by the US Census Bureau during the pandemic’s first year and are not included.

Figure 3 shows that the rate of individuals reporting multiple forms of coverage while enrolled in Medicaid increased during the study period. The share of those with self-reported Medicaid who also reported private coverage rose from 13.9% in 2019 to 16.1% in 2022, reflecting a 0.8 percentage point increase in nongroup coverage (including Marketplace insurance) and a 1.7 percentage point increase in ESI. Meanwhile, the share of Medicaid enrollees (as measured by survey data) who also had Medicare was essentially stable between 2019 and 2022.

## Discussion

The uninsured rate declined considerably from 2019 to 2022, with a 1.2 percentage point reduction that corresponds to nearly 4 million more US residents with insurance in 2022 than 2019. Various policies likely played a role in this coverage expansion during the pandemic, including the American Rescue Plan’s more generous subsidies for Marketplace coverage starting in 2021,^16^ enhanced government outreach efforts, and several recent state Medicaid expansions.^5^

In addition, the continuous coverage provision prevented states from removing people from Medicaid during the public health emergency, even if they would otherwise have lost eligibility. Official CMS administrative enrollment in Medicaid increased sharply between 2019 and 2022, consistent with this requirement. However, this growth in Medicaid enrollment far outstripped changes in survey-reported Medicaid and the uninsured rate. The unadjusted decline in uninsurance during this period was roughly one-fourth of the size of the growth in administrative Medicaid enrollment during the pandemic, or one-fifth in state-based regression models. Survey-based rates of Medicaid growth were also far smaller than administrative figures, especially for children and those not living in recent Medicaid expansion states.

Previous research has documented this survey undercount of Medicaid enrollment preceding and during the early pandemic.^8,17^ In part, this may reflect confusion among some beneficiaries about whether Medicaid-managed care is private insurance or Medicaid; the ACS questions do not include information on state program names or Medicaid-managed care plans.^18^ Research on federal surveys indicate that Medicaid beneficiaries more commonly mistakenly report having another form of coverage than report being uninsured.^19^

However, data from this study show that the Medicaid undercount worsened particularly in 2022, the last year before the end of the continuous coverage provision. There are several potential reasons for this worsening disconnect between survey and administrative data.

First, many people who maintained Medicaid enrollment throughout 2020 to 2022 may not have realized that their coverage continued. Typically, Medicaid eligibility only lasts a year and then must be renewed; transitions in and out of coverage are quite common.^20,21,22^ It may be that many beneficiaries—if they had not been contacted by the state to renew their coverage or were confused by paperwork they did receive—assumed their coverage had lapsed. Surveys conducted during the unwinding support the notion that many individuals in the US are confused about the pandemic-era policy.^23^ Notably, self-reported Medicaid rates diverged sharply from administrative Medicaid enrollment between 2021 and 2022, when the survey share of people reporting Medicaid essentially did not change, even as CMS Medicaid enrollment grew by 1.8 percentage points, or roughly 6 million people. This suggests that confusion or lack of awareness of ongoing Medicaid coverage may have gotten worse farther into the pandemic. Qualitative research would be helpful to illuminate beneficiaries’ perceptions and understanding of their coverage during the pandemic.

Second, some beneficiaries may have acquired other insurance in the meantime, particularly private insurance, further attenuating the relationship between administrative Medicaid coverage and uninsured rates. We detected a 2.2 percentage point increase in rates of private insurance among those who also reported having Medicaid, consistent with a recent government report on ESI among Medicaid beneficiaries and a progress report on Utah’s unwinding.^24,25^ This likely underestimates the true prevalence of people holding Medicaid and other insurance simultaneously, as some survey respondents may have only reported their non-Medicaid coverage.

The extent of the Medicaid undercount varied widely by state, though the undercount worsened in every single state during the 2019 to 2022 period. Variation in use of Medicaid-managed care, program outreach efforts, and population demographics may all contribute to variation in the extent of the state-level undercount.^18,26^ The strongest association between CMS Medicaid enrollment growth and declining uninsurance occurred in recent expansion states, likely reflecting uninsured people newly gaining Medicaid eligibility during new expansions, a particularly salient coverage change.

Growth in survey-reported Medicaid rates were larger among groups at higher risk of losing Medicaid eligibility (such as those with incomes slightly above the FPL) and therefore more likely to benefit from the continuous coverage provision. Nonetheless, even among these groups, the increases in self-reported Medicaid coverage were relatively modest, particularly from 2021 to 2022.

Confusion about coverage is not just an issue of survey measurement and projecting future coverage. The growing disconnect between Medicaid administrative enrollment and survey-reported coverage suggest a missed opportunity to promote continuity of care during the pandemic, as some beneficiaries appeared not to understand that their Medicaid coverage had remained in place for more than 2 years without requiring an eligibility redetermination. Given research evidence that changes in coverage—so-called “churning”—has numerous negative financial and health-related effects,^27,28^ future policy efforts to promote continuous coverage need to be clearly communicated to patients. For instance, starting in 2024, all states are required under federal law to provide 12 months of continuous coverage to children enrolled in Medicaid and the Children’s Health Insurance Program, meaning that they cannot lose their insurance mid-year even if their families experience a change in income or other circumstances.^29^ Similarly, more than 45 states have adopted an option under the American Rescue Plan to provide 12 months of continuous Medicaid eligibility to postpartum individuals,^30^ a marked change from the previous situation, in which more than half of all women experienced a coverage gap in the postpartum year.^21^ For these policies to produce their intended effects of improved continuity of care, beneficiaries need to understand the changes in the law and its effect on their coverage.

In addition, states continued to pay monthly capitation payments for beneficiaries in Medicaid-managed care plans, even if those beneficiaries did not realize that they were still in the program. Future research should explore the utilization patterns of such beneficiaries and the financial implications for state Medicaid spending. Lastly, these findings should not excuse states from their ongoing responsibility to reduce potential harms of the unwinding process and minimize coverage losses among people likely still eligible for Medicaid without alternative insurance.

### Limitations

This study has several limitations. The ACS does not include state-specific information or managed care plan names that might have helped more beneficiaries in Medicaid identify their coverage. We were not able to assess administrative enrollment by race and ethnicity, given lack of comprehensive public data from CMS that could be compared with survey data. Finally, the state-level regression models were only correlational and cannot provide any causal inferences tying the observed coverage changes to any particular policy.

## Conclusions

Results of this cross-sectional study have important implications for understanding patient perceptions of their insurance during the pandemic’s unprecedented continuous coverage provision and how that coverage may change during the unwinding period. As of February 2024, more than 16 million people had been disenrolled from Medicaid due to the unwinding process^7^; there are concerns that this disenrollment will reverse recent historic coverage gains, but currently available ACS data do not yet capture the effect of the unwinding process. The present analyses suggest that Medicaid disenrollment may not translate directly to increases in self-reported Medicaid loss for many individuals in the US, in part because an increasing number of beneficiaries may have alternative sources of coverage, but also because not all enrollees understood the continuity of their Medicaid benefits in the first place. Future policies to promote continuous eligibility in Medicaid should be paired with adequate beneficiary education if they are to achieve their goals of improved coverage and care.

## Notes

Supplement 1.
eMethods
**eTable 1.** Characteristics of Study Participants (N=12,506,584) **eTable 2.** State-Level Estimates of Coverage as a Share of the Population, Administrative versus Survey Data, 2019 vs. 2022 **eTable 3.** Survey-Reported Medicaid Coverage Changes for Groups at Risk for Eligibility Loss, 2019 to 2022 **eFigure.** State-Level Association Between Changes in Administrative Medicaid Enrollment Rates and Self-Reported Uninsured Rates (2019 vs. 2022), by Medicaid Expansion Timing

Supplement 2. Data Sharing Statement
