# Errors in Table 2 and eTables 1 and 3

**DOI:** 10.1001/jamahealthforum.2024.3865

**Published:** 2024-10-18

The Original Investigation titled “Survey-Reported Coverage in 2019-2022 and Implications for Unwinding Medicaid Continuous Eligibility,”^1^ published online April 5, 2024, was corrected to fix data errors in Table 2 and eTables 1 and 3 in Supplement 1, as well as related text in the Results section. The corrected results modestly affect the coverage estimates for income-based subgroups but do not affect the study’s main findings or conclusions. This article was corrected online.
