# Supplementary material for: Survey-Reported Coverage in 2019-2022 and Implications for Unwinding Medicaid Continuous Eligibility
Source: JAMA Health Forum. 2024 Apr 5;5(4):e240430. doi: 10.1001/jamahealthforum.2024.0430 (PMC10998158; doi:10.1001/jamahealthforum.2024.0430)
Supplement: Supplement 1. — eMethods eTable 1. Characteristics of Study Participants (N=12,506,584) eTable 2. State-Level Estimates of Coverage as a Share of the Population, Administrative versus Survey Data, 2019 vs. 2022 eTable 3. Survey-Reported Medicaid Coverage Changes for Groups at Risk for Eligibility Loss, 2019 to 2022 eFigure. State-Level Association Between Changes in Administrative Medicaid Enrollment Rates and Self-Reported Uninsured Rates (2019 vs. 2022), by Medicaid Expansion Timing [file jamahealthforum-e240430-s001.pdf]

Supplemental Online Content

McIntyre A, Smith RB, Sommers BD. Survey-reported coverage in 2019-2022 and implications for unwinding Medicaid continuous eligibility. *JAMA Health Forum*. 2024;5(4):e240430. doi:10.1001/jamahealthforum.2024.0430

eMethods
eTable 1. Characteristics of Study Participants (N=12,506,584)
eTable 2. State-Level Estimates of Coverage as a Share of the Population, Administrative versus Survey Data, 2019 vs. 2022
eTable 3. Survey-Reported Medicaid Coverage Changes for Groups at Risk for Eligibility Loss, 2019 to 2022
eFigure. State-Level Association Between Changes in Administrative Medicaid Enrollment Rates and Self-Reported Uninsured Rates (2019 vs. 2022), by Medicaid Expansion Timing

This supplemental material has been provided by the authors to give readers additional information about their work.

© 2024 McIntyre A et al. *JAMA Health Forum.*

eMethods
American Community Survey Health Insurance Questions

The study's measures of health insurance coverage are based upon responses to the following survey questions in the ACS:

"Is this person CURRENTLY covered by any of the following types of health insurance or health coverage plans?

a. Insurance through a current or former employer or union (of this person of another family member)
b. Insurance purchased directly from an insurance company (by this person or another family member)
c. Medicare, for people 65 and older, or people with certain disabilities
d. Medicaid, Medical Assistance, or any kind of government-assistance plan for those with low incomes or a disability
e. TRICARE or other military health care
f. VA (including those who have ever used or enrolled for VA health care)
g. Indian Health Service
h. Any other type of health insurance or health coverage plan

Note that anyone responding "no" to all items above other than "question g" (since Indian Health Service is not considered health insurance by the U.S. Census Bureau) is classified as "Uninsured."

High-Risk Eligibility Groups

We assessed changes in self-reported coverage for several subgroups who may have been more likely to benefit from the continuous coverage provision – groups for whom loss of

© 2024 McIntyre A et al. *JAMA Health Forum.*

Medicaid eligibility would be more common than other populations, and who therefore may have been able to remain in Medicaid at higher rates in 2022 than previous years due to the continuous coverage provision. These groups are: 1) childless adults in expansion states with incomes between 138 and 200% of the Federal Poverty Level (FPL), who would generally lose eligibility based on their income being above the Medicaid expansion threshold; 2) children with incomes between 250 and 400% FPL, who in many states would lose eligibility for Medicaid or CHIP; 3) mothers of children ages 1 or younger, who are at high risk for post-partum loss of Medicaid eligibility; and 4) adults 19 years of age, who often lose Medicaid eligibility by virtue of moving from childhood status to adult status (with lower associated income thresholds).

© 2024 McIntyre A et al. *JAMA Health Forum.*

eTable 1. Characteristics of Study Participants (N=12,506,584)

Characteristic	Survey-Weighted Percent (Number)
Male	49.4% (6,147,857)
Female	50.6% (6,358,727)
American Indian and Alaska Native (Non-Hispanic)	0.6% (108,919)
Asian-American, Native Hawaiian, and Pacific Islander (Non-Hispanic)	5.9% (743,890)
Black (Non-Hispanic)	12.0% (1,122,246)
Hispanic	18.7% (1,851,095)
White (Non-Hispanic)	58.6% (8,148,673)
Age 0-18	23.4% (2,598,730)
Age 19-64	59.7% (7,194,833)
Age 65 and Older	16.9% (2,713,021)
Income 0-138% FPL	25.3% (3,082,226)
Income 139-250% FPL	18.2% (2,143,085)
Income 251%-400% FPL	19.0% (2,346,003)
Income >400% FPL	37.5% (4,935,270)

Note: Income was defined by the health insurance unit, as a percentage of the Federal Poverty Level.

© 2024 McIntyre A et al. *JAMA Health Forum.*

eTable 2. State-Level Estimates of Coverage as a Share of the Population, Administrative versus Survey Data, 2019 vs. 2022

State	2019 (%)				2022 (%)			
	Survey Uninsured	Survey Medicaid	Administrative Medicaid	Undercount (Admin – Survey)	Survey Uninsured	Survey Medicaid	Administrative Medicaid	Undercount (Admin – Survey)
AL	10.1%	19.6%	18.7%	-0.9%	8.9%	21.0%	22.4%	1.4%
AK	11.5%	21.0%	30.1%	9.1%	10.5%	22.8%	35.6%	12.8%
AZ	11.7%	21.0%	23.5%	2.5%	10.6%	21.0%	30.2%	9.2%
AR	9.6%	26.5%	26.5%	0.0%	9.2%	27.4%	33.2%	5.7%
CA	8.0%	25.6%	29.8%	4.2%	6.6%	27.0%	35.1%	8.1%
CO	8.1%	17.0%	22.4%	5.4%	7.3%	18.3%	28.2%	9.9%
CT	5.9%	22.0%	24.1%	2.1%	5.2%	22.7%	27.1%	4.5%
DE	7.2%	20.5%	23.8%	3.3%	5.7%	19.8%	28.5%	8.7%
DC	3.7%	24.9%	36.0%	11.2%	3.1%	23.5%	42.5%	19.0%
FL	13.6%	17.5%	17.0%	-0.5%	11.5%	18.0%	21.2%	3.2%
GA	14.0%	17.3%	17.3%	0.0%	12.2%	18.4%	22.0%	3.6%
HI	4.2%	17.5%	23.2%	5.8%	3.6%	19.5%	31.3%	11.7%
ID	10.5%	15.8%	15.0%	-0.8%	8.1%	20.8%	22.1%	1.3%
IL	7.4%	18.7%	22.3%	3.6%	6.7%	20.6%	29.2%	8.6%
IN	9.0%	18.3%	19.4%	1.2%	6.9%	21.0%	28.5%	7.5%
IA	4.9%	19.7%	21.4%	1.7%	4.2%	20.8%	25.9%	5.1%
KS	9.4%	14.2%	12.9%	-1.3%	8.8%	14.5%	16.7%	2.2%
KY	6.7%	26.0%	29.2%	3.2%	5.8%	28.6%	34.9%	6.2%
LA	9.5%	29.6%	32.2%	2.6%	7.4%	32.5%	40.4%	7.9%
ME	8.1%	20.5%	19.0%	-1.5%	6.5%	20.2%	25.6%	5.4%
MD	6.0%	19.1%	21.9%	2.8%	6.1%	20.0%	26.6%	6.6%
MA	3.1%	22.3%	23.1%	0.8%	2.4%	23.7%	27.5%	3.8%
MI	6.0%	22.0%	23.2%	1.2%	4.8%	23.6%	29.5%	5.9%
MN	4.9%	17.3%	18.6%	1.3%	4.5%	18.6%	23.3%	4.6%
MS	13.7%	24.2%	20.8%	-3.4%	11.5%	24.2%	25.5%	1.3%
MO	10.4%	14.9%	14.2%	-0.7%	8.7%	16.5%	21.4%	4.9%
MT	8.4%	21.4%	25.4%	4.0%	8.0%	21.7%	28.0%	6.3%
NE	8.1%	12.9%	12.8%	-0.2%	6.7%	16.1%	19.0%	2.9%
NV	11.8%	18.0%	20.5%	2.5%	11.3%	20.9%	27.2%	6.2%
NH	6.5%	13.7%	13.3%	-0.4%	4.7%	13.6%	17.4%	3.8%
NJ	8.0%	17.1%	19.4%	2.3%	6.9%	18.8%	23.2%	4.4%
NM	10.2%	32.8%	35.2%	2.4%	8.5%	33.6%	41.5%	7.9%
NY	5.4%	26.1%	31.3%	5.2%	4.9%	28.8%	36.8%	8.0%
NC	11.4%	18.1%	16.7%	-1.4%	9.5%	18.7%	21.2%	2.6%
ND	7.3%	12.5%	11.9%	-0.7%	6.6%	12.6%	16.0%	3.5%
OH	6.9%	20.5%	22.6%	2.0%	6.0%	21.7%	27.8%	6.1%
OK	15.3%	17.3%	18.4%	1.1%	12.3%	22.3%	30.5%	8.2%
OR	7.3%	21.1%	23.4%	2.3%	6.1%	23.6%	31.2%	7.6%
PA	6.1%	20.6%	23.5%	2.9%	5.6%	21.8%	27.8%	6.0%
RI	4.2%	20.8%	28.4%	7.6%	4.0%	22.3%	32.3%	10.1%

© 2024 McIntyre A et al. *JAMA Health Forum.*

**SC**	11.0%	18.9%	20.5%	1.5%	9.2%	19.7%	24.0%	4.2%
**SD**	9.7%	13.4%	12.5%	-1.0%	8.2%	13.7%	15.4%	1.7%
**TN**	10.6%	19.7%	21.3%	1.6%	9.7%	19.2%	24.6%	5.4%
**TX**	18.9%	16.0%	14.6%	-1.4%	17.0%	16.9%	18.4%	1.5%
**UT**	9.8%	9.6%	9.5%	-0.1%	8.2%	10.8%	13.7%	2.9%
**VT**	4.3%	24.0%	24.8%	0.8%	3.8%	22.7%	29.2%	6.6%
**VA**	8.1%	13.8%	15.6%	1.7%	6.6%	16.9%	22.2%	5.3%
**WA**	6.6%	20.1%	22.7%	2.6%	6.0%	20.9%	27.2%	6.3%
**WV**	7.0%	27.0%	28.8%	1.8%	6.6%	28.3%	35.5%	7.2%
**WI**	6.0%	16.5%	17.8%	1.3%	5.5%	18.4%	23.4%	5.0%
**WY**	12.4%	11.9%	9.8%	-2.1%	11.7%	12.1%	13.5%	1.4%

© 2024 McIntyre A et al. *JAMA Health Forum.*

eTable 3. Survey-Reported Medicaid Coverage Changes for Groups at Risk for Eligibility Loss, 2019 to 2022

Group	2019 Medicaid	2021 Medicaid	2021 vs. 2019	2022 Medicaid	2022 vs. 2021
Childless Adults 19-64, Income 138-200% FPL in Expansion States	24.2%	26.9%	+2.7 pp	29.2%	+2.3 pp
Parents 19-64, Incomes 100-200% FPL in Non-Expansion States	15.6%	20.8%	+5.2 pp	22.0%	+1.2 pp
Children 0-18, Incomes 250-400% FPL	18.5%	22.6%	+4.1 pp	23.5%	+0.9 pp
19 Year-Old Adults	18.5%	22.6%	+4.1 pp	23.5%	+0.9 pp
Mothers of Children Ages 0-1	27.4%	28.7%	+1.3 pp	28.5%	-0.2 pp
Adults Ages 19-64, with Employer-Sponsored Insurance	2.0%	2.6%	+0.6 pp	2.7%	+0.0 pp

© 2024 McIntyre A et al. *JAMA Health Forum.*

eFigure 1. State-Level Association Between Changes in Administrative Medicaid Enrollment Rates and Self-Reported Uninsured Rates (2019 vs. 2022), by Medicaid Expansion Timing

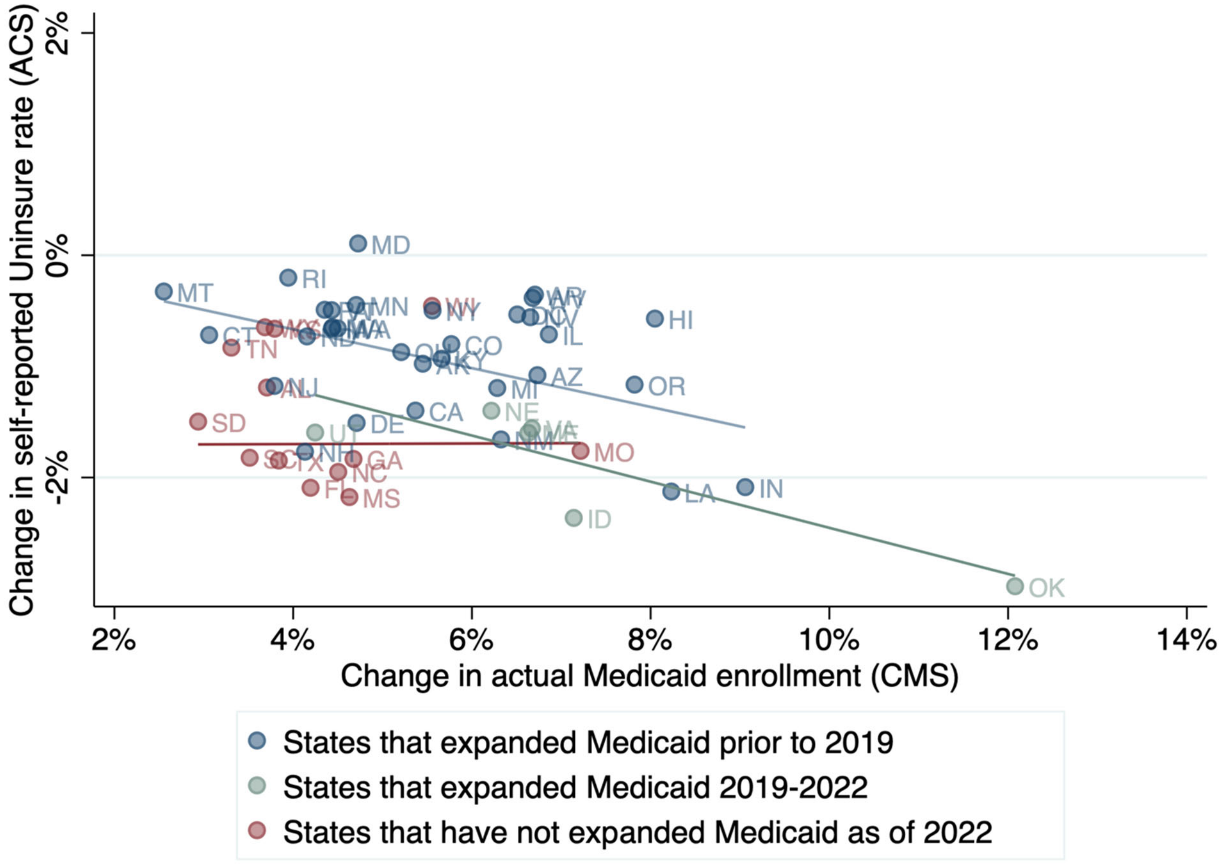

Regression coefficients (95% CI) were as follows:

- Recent expansion states, ß=-0.21 (-0.32 to -0.10)
- Older expansion states, ß=-0.18 (-0.32 to -0.03);
- Non-expansion states, ß=0.03 (-0.32 to 0.33)

Notes: Changes refer to state-level percentage-point changes between 2019 and 2022 in each outcome. Lines are the fitted linear regression estimates for a state-level model (n=51 for all states plus Washington DC); letters refer to state abbreviations and regressions are weighted by state population).

© 2024 McIntyre A et al. *JAMA Health Forum.*
