# Supplementary material for: Survey-Reported Coverage in 2019-2022 and Implications for Unwinding Medicaid Continuous Eligibility
Source: JAMA Health Forum. 2024 Apr 5;5(4):e240430. doi: 10.1001/jamahealthforum.2024.0430 (PMC10998158; doi:10.1001/jamahealthforum.2024.0430)
Supplement: Supplement 2. — Data Sharing Statement [file jamahealthforum-e240430-s002.pdf]

Data Sharing Statement

McIntyre. Survey-Reported Coverage in 2019-2022 and Implications for Unwinding Medicaid Continuous Eligibility. *JAMA Health Forum*. Published April 05, 2024.
doi:10.1001/jamahealthforum.2024.0430

Data
Data available: All data used in this analysis are publicly available. Additional information on data analysis is available from the authors upon request.
